# Mining and analysis of dizziness adverse event signals in postoperative analgesia patients based on the FDA adverse event reporting system database

**DOI:** 10.3389/fphar.2025.1488469

**Published:** 2025-02-13

**Authors:** Fengqi Zhou, Haiou He, Jing Gao, Zhen Zhang

**Affiliations:** ^1^ Department of Anesthesiology, Xiangyang No. 1 People’s Hospital, Hubei University of Medicine, Xiangyang, Hubei, China; ^2^ Department of Anesthesiology, Suizhou Hospital, Hubei University of Medicine, Suizhou, Hubei, China

**Keywords:** dizziness, postoperative analgesia, FAERS, drug safety, signal detection

## Abstract

**Objective:**

This study aimed to explore the association between drugs used in postoperative anesthesia patients and postoperative dizziness using the U.S. Food and Drug Administration’s Adverse Event Reporting System (FAERS) database, along with other risk factors for dizziness.

**Methods:**

Using the FAERS database, we retrospectively analyzed dizziness cases reported between 2004 and the third quarter of 2023. We analyzed the relationship between drugs during postoperative anesthesia and the risk of postoperative dizziness, and conducted subgroup analysis according to age, sex and other factors. Signal detection was further performed using the reported odds ratio (ROR) method to identify medications significantly associated with an increased risk of postoperative dizziness.

**Results:**

A total of 166,292 dizziness case reports were obtained, with 128 cases specifically related to postoperative analgesia. The number of dizziness reports has been increasing yearly, with a higher concentration of cases among individuals aged 18–85 years, predominantly in female patients. The analysis identified that amitriptyline, clonazepam, and ketamine were significantly associated with an increased risk of dizziness, with RORs of 34.91, 17.39, and 7.37, respectively. Subgroup analyses revealed variations in the relative risk of dizziness based on sex and age groups. Ketamine may be associated with higher risk of dizziness in the adult male subgroup.

**Conclusion:**

The results of this study suggest that specific medications used by patients with postoperative analgesia are associated with an increased risk of postoperative dizziness. Future studies should further validate this finding and explore other potential risk factors.

## Introduction

Dizziness is a common but often ignored issue in clinical practice, especially in postoperative settings (Neuhauser, 2016). It not only prolongs patient recovery times and increases hospital costs but also elevates the risk of falls, which may lead to further complications or injury (Kerber and Newman-Toker, 2015; Bösner et al., 2018; Barin and Dodson, 2011). Additionally, dizziness can be an indicator of underlying, undiagnosed complications such as vestibular dysfunction or metabolic imbalances (Kerber and Newman-Toker, 2015; Bösner et al., 2018; Barin and Dodson, 2011). While numerous factors are known to contribute to dizziness, including nervous system disorders and nutritional deficiencies, the specific relationship between drug use and the occurrence of dizziness has not been thoroughly explored (Newman-Toker and Edlow, 2015).

Postoperative healthcare is critical to maintaining the patient’s physiological status, alleviating symptoms, and preventing complications (Jin et al., 2020; Cao et al., 2017). During this period, medications, particularly analgesics and anesthetic agents, are commonly administered. However, the use of these drugs can also lead to adverse reactions, including dizziness, which can indirectly affect the patient’s recovery and overall wellbeing (Derebery, 1999; Eggers and Staab, 2024). While several studies have investigated the relationship between various medications and postoperative adverse effects, systematic investigations focusing on the association between postoperative anesthetic drug use and dizziness remain limited. Furthermore, with the continuous introduction of new medications into clinical practice, the safety profiles of these drugs are still under observation, highlighting the need for updated research on this topic.

Signal detection in pharmacovigilance refers to the identification of potential associations between a drug and an adverse event, indicated by disproportionality in the number of reported adverse events (Edwards and Aronson, 2000; Bate et al., 2014). The detection of a signal marks the first step in pharmacovigilance, which is followed by further investigations to determine the likelihood of a causal relationship through methods such as disproportionality analysis and clinical evaluation. The U.S. Food and Drug Administration (FDA) Adverse Event Reporting System (FAERS) is a public database that compiles a vast array of adverse drug events reported in clinical practice. It serves as a valuable resource for investigating potential associations between drug use and adverse events such as dizziness (Xia et al., 2023; Neha et al., 2021). Previous research using FAERS has helped to identify adverse drug reactions (ADRs) and their clinical significance (Gustafsson et al., 2023; Villa-Zapata et al., 2022). However, few studies have specifically explored the relationship between postoperative anesthetic drug use and dizziness through this database.

Given these gaps in the literature, this study aims to systematically analyze FAERS data to explore the relationship between postoperative anesthetic drug use and the risk of dizziness. By identifying specific drugs or drug categories associated with an increased risk of postoperative dizziness, we hope to contribute to a better understanding of the factors influencing postoperative recovery. This research aims to provide insights that could help improve postoperative outcomes, ultimately enhance recovery quality, and improve patients’ quality of life.

## Methods

### Data sources

The data for this study was from the FAERS database. OpenVigil 2.0 (Orzetti et al., 2022; Soldatos et al., 2022) and AERSMine online tool (Sarangdhar et al., 2016) were used for analysis. FAERS is a voluntary reporting system with wide coverage and large data volume that can provide real-world data on adverse drug reactions (Alatawi and Hansen, 2017). In this study, all data extraction and processing are in compliance with the data protection and privacy policies of the respective databases.

### Research design

This study adopted a retrospective cohort study design. First, we defined the case selection criteria: dizziness as a report indicating any subjective complaint of dizziness in association with analgesic use after surgery. The role of the drug was primary suspect. Subsequently, relevant data was extracted from the FAERS database, including patient age, sex, and drug name, reporting area, reporter type, final outcome and other related information.

**FIGURE 1:**
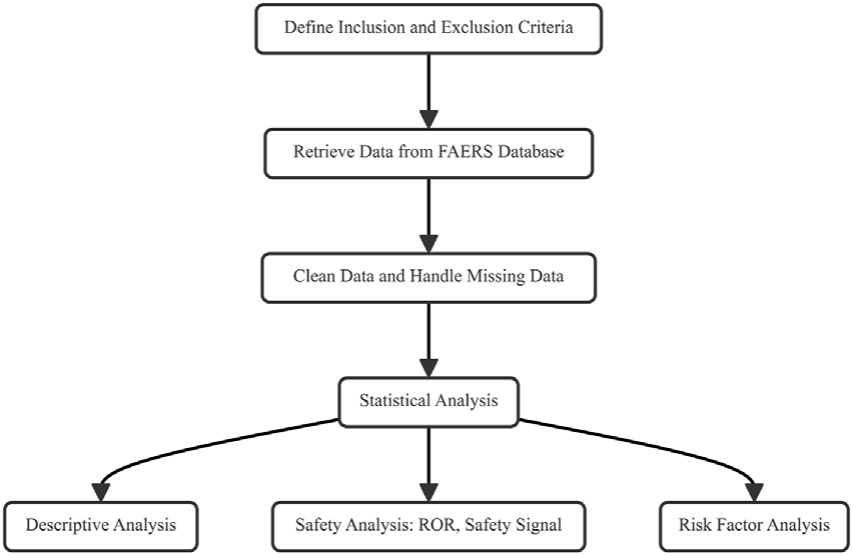
Study flow chart.

The data screening and processing process was as follows (Neuhauser, 2016): excluded cases with incomplete data or duplicate reports, and limit the age to 18 years and above (Kerber and Newman-Toker, 2015); standardized drug names to enable the identification and analysis of specific drugs or drug classes (Bösner et al., 2018); we used absolute count, safety signal, relative risk, reporting odds ratio (ROR) and other methods to conduct preliminary signal and safety signal detection to identify potential drugs related to postoperative dizziness (Figure 1).

### Statistical analysis

The statistical analysis of this study had three sections: descriptive statistics, signal detection methods, and subgroup analysis. Descriptive statistics were used to outline the baseline characteristics of the study cohort, including patient demographics, medication use, and occurrence of postoperative dizziness. Signal detection methods such as absolute count, safety signal, relative risk, ROR, and bayesian confidence propagation neural network (BCPNN) were used to identify drug signals associated with increased risk of dizziness. Among them, safety signal and relative risk are generated by AERSMine online analysis (Sarangdhar et al., 2016). When the absolute count is greater than or equal to 2, the safety signal is greater than 0, and the relative risk is greater than 2, the drug is considered to be related to dizziness (Hauben and Zhou, 2003). The ROR and its 95% confidence interval (CI) were calculated. If the lower limit of the 95% CI of the ROR was greater than 1, it was considered that there was a potential positive association between the drug and postoperative dizziness. The BCPNN method was used to detect signals by applying Bayesian statistics. This method is implemented using the pvm package in R, which allows us to calculate BCPNN signal for drug-adverse event associations. BCPNN is a more sensitive yet complex method that relies on Bayesian inference to estimate the strength of the signal, balancing sensitivity and specificity. Signal Detection Criteria: We used the following criteria to identify potential adverse drug reactions (ADR) signals using BCPNN: Report count ≥3: A drug-event combination must have at least three reports. IC 95% Confidence Interval Lower Limit >0: The 95% confidence interval for the Information Component (IC) must have its lower limit greater than 0. Strength of Signal: The strength of the signal suggests the degree of association between a drug and an ADR. A positive signal, with a sufficiently strong IC value and a lower 95% CI > 0, is considered a potential ADR signal. To assess the relationship between a drug or drug class and the risk of postoperative dizziness in a specific population, we introduced subgroup analyzes by sex and age. Specifically, subgroups were defined based on key characteristics such as sex, drug types, and patient demographics. We used OpenVigil 2.1 (https://openvigil.sourceforge.net/#) as our primary tool for signal detection and subgroup analysis. This tool allowed us to extract and analyze data from the FAERS database, with the sample sizes for each subgroup being calculated based on the number of reports available within the tool. All statistical analyzes were performed using the statistical software Python (version 3.12) and/or R (4.4.1).

## Results

### Baseline characteristics of the included reports

**FIGURE 2:**
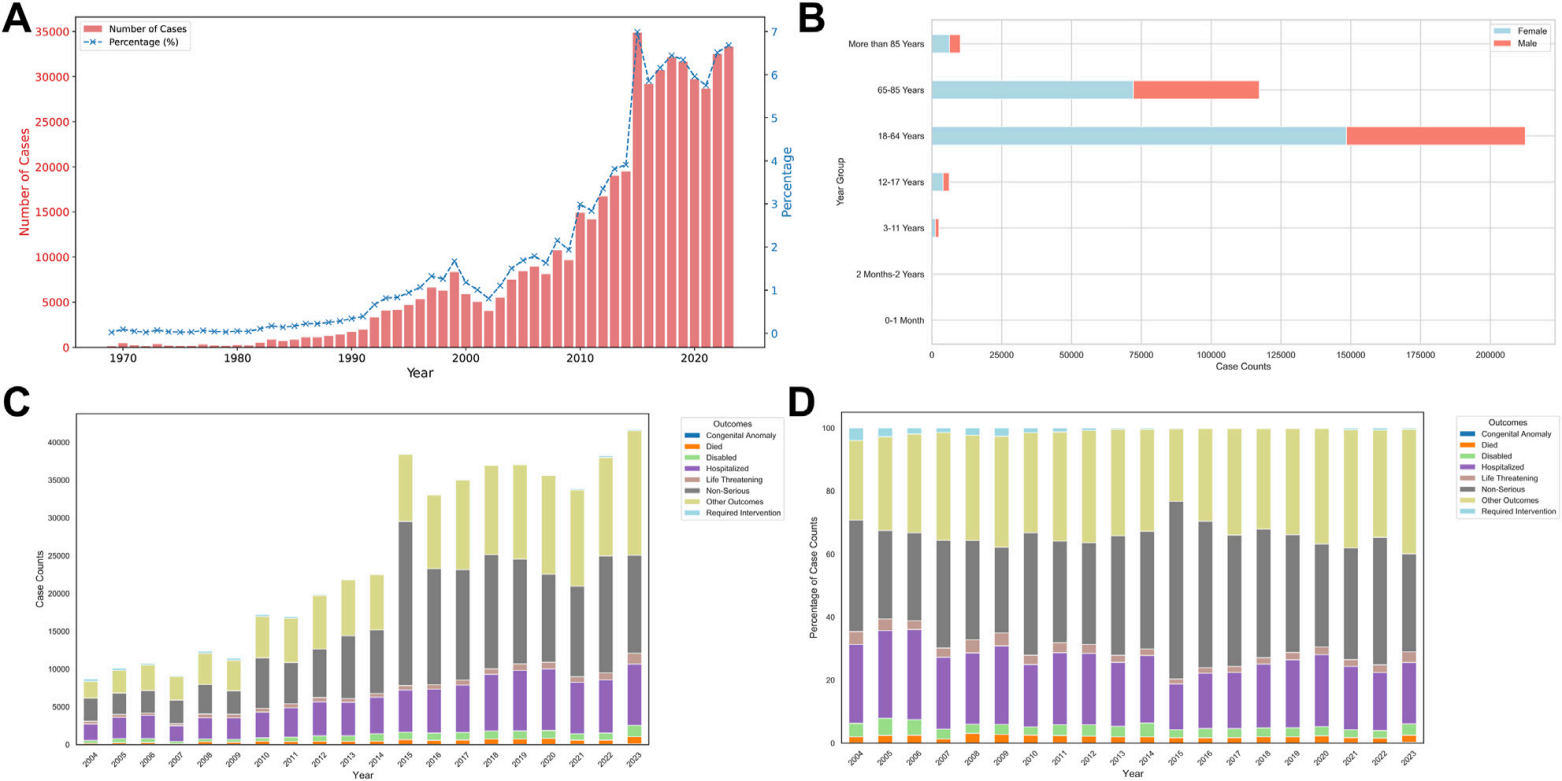
Baseline characteristics of the dizziness adverse event in postoperative analgesia patients. **(A)**, Count number of dizziness adverse event yearly; **(B)**, patients with dizziness in different age and sex groups; **(C)**, Absolute count of different patient outcomes grouped by years; **(D)**, Percentage of different patient outcomes grouped by years.

This study analyzed a total of 166,292 reports of dizziness adverse reaction from the FAERS database, including 128 reports of dizziness events during postoperative anesthesia. The absolute count of reported dizziness showed an upward trend year by year, reaching a peak in 2015 and then stabilizing at a high level (Figure 2A). Among all reported dizziness events, 61.92% (n = 308,892) were female, and the age distribution was concentrated between 18 and 85 years old (Table 1). This trend was particularly obvious among females (Figure 2B). In terms of outcomes, absolute count (Figure 2C) and percentage (Figure 2D) for the non-serious patients were the most common. In the setting of absolute count, the number of patients requiring hospitalization and disability was on the rise in recent years (Figure 2C). Among patients who reported dizziness, classified by generic name, the number of drugs involved was 5,608. Among them, the top five most commonly reported drugs were Adalimumab, Pregabalin, Etanercept, Teriparatide, and Sacubitril\Valsartan, with a reported number of 11,492 (2.30%), 9,062 (1.82%), 8,733 (1.75%), 8,382 (1.68%), and 8,063 (1.62%), respectively. Among patients who reported postoperative anesthesia combined with dizziness, the top five most commonly reported drugs included Bupivacaine (n = 9, 13.43%), Acetaminophen\Hydrocodone Bitartrate (n = 7, 10.45%), Morphine (n = 4, 5.97%), Lidocaine (n = 4, 5.97%), and Adalimumab (n = 3, 4.48%), respectively.

**TABLE 1:** Baseline characteristics of the included patients with dizziness.

Variables	Category	Number of cases	Percentage
Age	0–1 Month	98	0.02%
	2 Months–2 Years	279	0.06%
	3–11 Years	2,545	0.51%
	12–17 Years	6,286	1.26%
	18–64 Years	213,999	42.90%
	65–85 Years	118,117	23.68%
	More than 85 Years	10,276	2.06%
	Not Specified	147,276	29.52%
Sex	Female	308,892	61.92%
	Male	156,002	31.27%
	Not Specified	33,982	6.81%
Reporter	Healthcare Professional	177,851	35.65%
	Consumer	292,090	58.55%
	Other	2	0.00%
	Not Specified	28,933	5.80%

### Drug safety signal detection results

**FIGURE 3:**
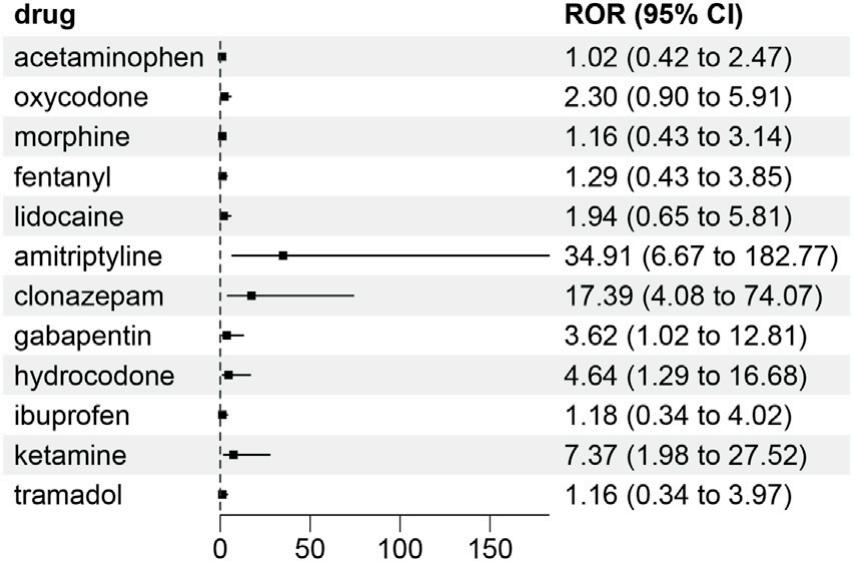
Forest plot of drug safety signal detection results in terms of reported odds ratio.

We performed ROR analysis on 77 drugs used for patients with postoperative anesthesia (Figure 3). The results showed that amitriptyline (ROR = 34.91, 95% CI: 6.67–182.77; *P* < 0.01), clonazepam (ROR = 17.39, 95% CI: 4.08–74.07; *P* < 0.01), ketamine (ROR = 7.37, 95% CI: 1.98–27.52; *P* < 0.01), hydrocodone (ROR = 4.64, 95% CI: 1.29–16.68; *P* = 0.2), and gabapentin (ROR = 3.62, 95% CI: 1.02–12.81; *P* = 0.2) were associated with risk of postoperative dizziness events. Additionally, the BCPNN IC values for these drugs were as follows: amitriptyline (IC = 1.65, 95% CI lower = 0.81), clonazepam (IC = 1.51, 95% CI lower = 0.63), ketamine (IC = 1.21, 95% CI lower = 0.23), hydrocodone (IC = 0.98, 95% CI lower = −0.10), and gabapentin (IC = 0.83, 95% CI lower = −0.33). The lower limit of the IC’s 95% CI indicated the robustness of the signal for these drugs, particularly amitriptyline, clonazepam, and ketamine, which had positive IC values with their lower limits above 0, suggesting significant associations with postoperative dizziness.

**FIGURE 4:**
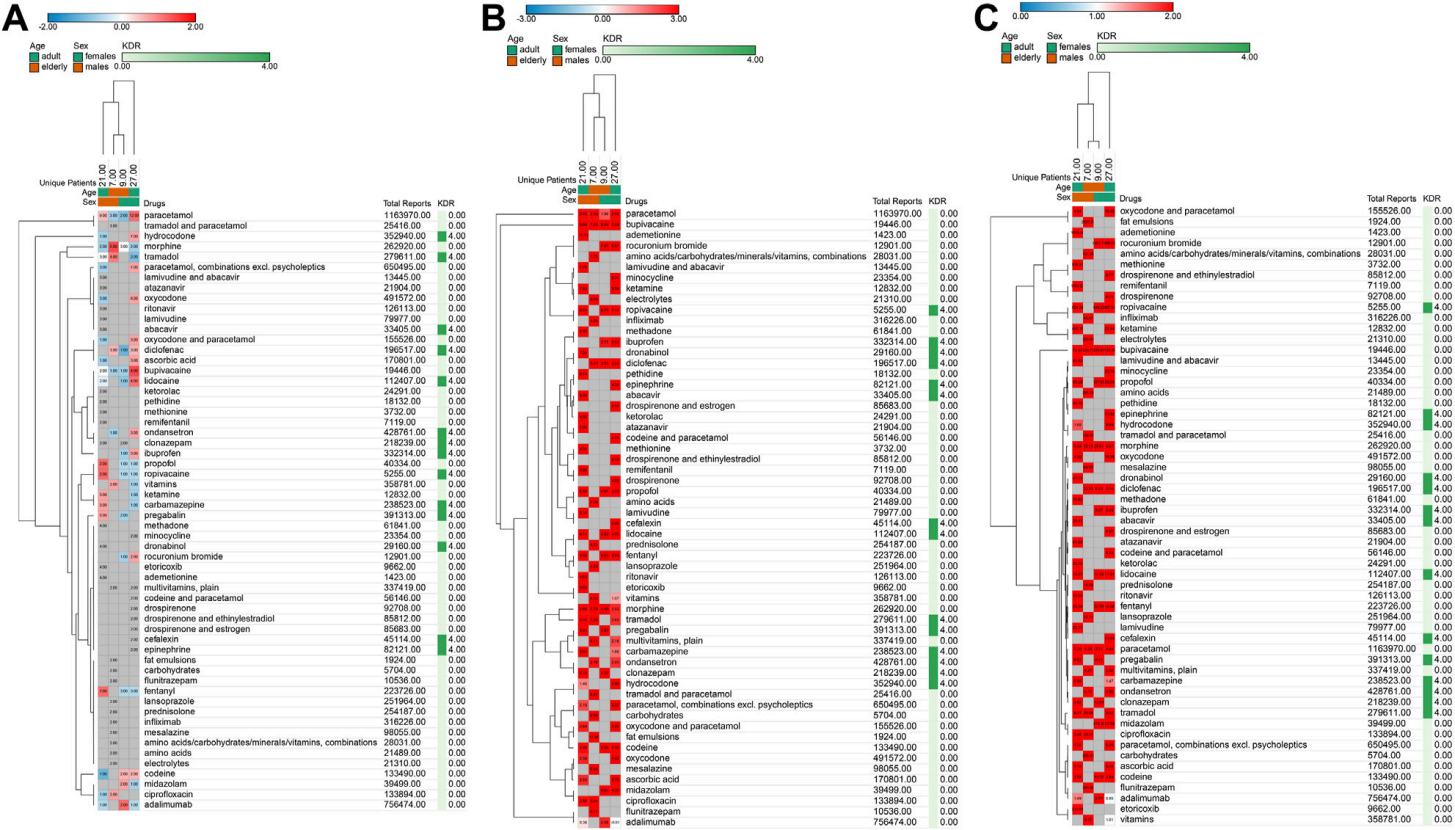
Heatmap presentation of quantitative safety metrics results based on sex and age groups **(A)**, absolute count; **(B)**, safety signal; **(C)**, relative risk.

Subsequently, we divided the patients into adult males (male case with age from 25 to 65 years), elderly males (male case with age above 65 years), adult females (female case with age from 25 to 65 years), and elderly females (female case with age above 65 years) according to age and sex for subgroup analysis (Figure 4). We conducted subgroup analysis on absolute count (Figure 4A), safety signal (Figure 4B), and relative risk (Figure 4C). The results showed that the five drugs with the highest relative risk values in the adult male subgroup were: Ademetionine, Remifentanil, Methionine, Ropivacaine and Ketamine, respectively. For the elderly male subgroup were: Fat emulsions, Carbohydrates, Flunitrazepam, Bupivacaine and Electrolytes, respectively. For the adult female subgroup: Rocuronium bromide, Ropivacain, Bupivacaine, Midazolam and Propofol, respectively. For elderly female subgroup: Rocuronium bromid, Ropivacain, Bupivacaine, Propofol and Midazolam, respectively. These results showed the drugs with higher relative risks in different age and sex subgroups, suggesting that safety differences related to age and sex should be considered.

**FIGURE 5:**
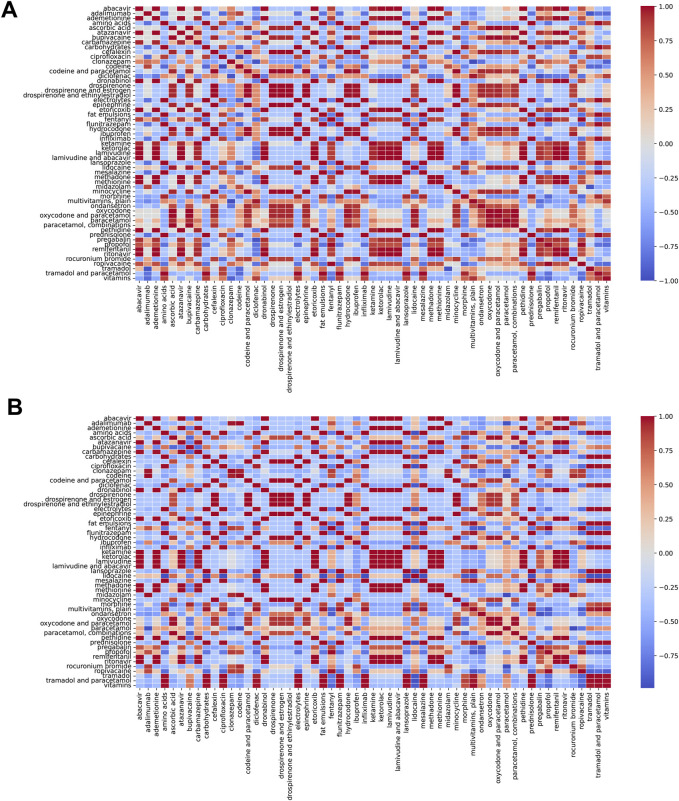
Correlation analysis of drugs interactions on the risk of dizziness postoperative analgesia patients **(A)**, Absolute count; **(B)**, relative risk.

We performed correlation analyzes on the absolute counts (Figure 5A) and relative risks (Figure 5B) of the involved drugs. The results showed that the top ten pairs of drugs with the strongest negative correlation and their correlation coefficients were as follows: the negative correlation coefficient of Rocuronium Bromide and Paracetamol was −0.99, for Paracetamol and Midazolam was −0.98, for Codeine and Paracetamol was −0.97, for Ciprofloxacin and Lidocaine was −0.97, for Adalimumab and Paracetamol was −0.96, for Lidocaine and Tramadol and Paracetamol was −0.96, for Pregabalin and Ondansetron was −0.95, for Lidocaine and Tramadol was −0.94, for Lidocaine and Diclofenac was −0.93, and Ropivacaine and Multivitamins, plain was −0.91, respectively.

## Discussion

This study analyzed data from the FAERS database and found that the use of opioids, nonsteroidal anti-inflammatory drugs, and specific nutritional drugs during postoperative anesthesia was associated with a significantly increased risk of postoperative dizziness. At the same time, there were significant differences in the risk of postoperative dizziness with regards to patients’ sex, age, and different types of drugs. These suggest that individual differences should be taken into account in the drug administration, thereby minimizing the risk of adverse reactions caused by postoperative drugs.

Our study identified significant trends in dizziness reports associated with various drug classes, with notable variations based on patient sex and age. These findings contribute to our understanding of the potential risk factors for dizziness in pharmacovigilance. However, it is important to interpret these results with caution. While we observed an increase in dizziness reports over time, this could reflect improved reporting practices or changes in drug usage patterns rather than an actual increase in adverse events. Further studies with controlled cohorts and better longitudinal data are needed to validate these trends.

Compared with other studies, our findings are consistent with those of several studies that also reported an association of opioids and NSAIDs with adverse effects of postoperative dizziness (Wang et al., 2023; Chassery et al., 2024; Shen et al., 2022). The study by Teng-Kuan Wang and colleges compared the clinical efficacy and adverse effects of a multimodal analgesic (MA) strategy involving peripheral nerve block (NB), periarticular injection (PAI), and intravenous patient-controlled analgesia (IVPCA), versus patient -controlled epidural analgesia (PCEA) for patients undergoing total knee arthroplasty (TKA) (Wang et al., 2023). The study found lower incidence of dizziness in the MA group compared to the PCEA group on the first postoperative day. PCEA can cause dizziness in some patients due to opioid medications, sympathetic blockade, or dural puncture. Opioid-related adverse effects like dizziness, nausea, vomiting, and respiratory depression remain major concerns. In addition, older patients often have more comorbidities, raising postoperative issues like dizziness (Wang et al., 2023). Another study aimed to assess the potential benefits of opioid-free anesthesia (OFA) over opioid-sparing anaesthesia (OSA) in day-case primary total hip arthroplasty (Chassery et al., 2024). The study involved 80 patients undergoing total hip arthroplasty under general anaesthesia. Patients received total intravenous anaesthesia with a laryngeal mask and multimodal analgesic regimen with non-opioid analgesics. Results showed pain scores being similar and low in all groups, and walking recovery time being comparable. Adverse events were sparse but not dizziness, which was more common in the OSA group (Chassery et al., 2024). The purpose of the study by Jiahong Shen et al. was to assess whether low-dose esketamine affected the patients’ *postpartum* depression following cesarean delivery (Shen et al., 2022). One group received esketamine (Group S) and the other for normal saline (Group L). The S group experienced a higher incidence of dizziness (P < 0.001) at 5 min, 15 min after dose than the L group, respectively. Even when leaving the operation room, the S group still experienced a higher incidence of dizziness (P < 0.001) than the L group (Shen et al., 2022). These suggest that postoperative anesthetic use may increase the risk of dizziness. However, our study further refines the relationship between these drugs and specific adverse events of postoperative dizziness and provides an analysis of safety signals through real-world data. Through the application of ROR signal detection method, this study revealed the association between the use of certain postoperative anesthetic drugs and postoperative dizziness, which has been rarely reported in the previous literature.

Through this study, we found that the use of postoperative anesthesia drugs may be improved in several aspects to reduce the risk of postoperative dizziness. First, the use of medications in the postoperative period requires consideration that certain medications, such as opioid analgesics, may increase the risk of postoperative dizziness. Opioid analgesics, commonly used for pain management, can induce dizziness through their central nervous system depressant effects. Opioids act on the mu-opioid receptors in the brain and spinal cord, leading to sedation and alterations in the vestibular system. Additionally, opioids can cause hypotension due to their vasodilatory effects. These may result in dizziness (Lambert, 2023). Secondly, the monitoring of adverse reactions in postoperative patients should be strengthened to promptly identify and manage possible adverse reactions, such as dizziness. Third, the results of this study indicate that patients of different sexs and ages have significant differences in the risk of dizziness after receiving different types of drug treatment. It is suggested that medication should be selected appropriately based on the patient’s baseline characteristics to minimize the risk of postoperative adverse reactions. In addition, the risk of dizziness is significantly reduced when certain medications are used together. This suggests that for high-risk patients who are prone to dizziness, preventive medication may be considered to reduce the risk of dizziness.

Critically ill patients are often at greater risk for a range of complications, including electrolyte imbalances, which could potentially contribute to dizziness independently of the treatment they receive. It is also important to consider the complexity of surgeries or interventions these patients undergo, which might result in both metabolic disturbances and systemic effects that influence the onset of dizziness. These associations may be influenced by the underlying illness, and the procedural complexity involved in critical care. Further research is needed to accurately assess the independent effects of fat emulsions and electrolyte abnormalities on dizziness, accounting for the confounding factors inherent in the intensive care unit environment.

This study has several limitations that must be considered when interpreting the results. First, the FAERS database relies on voluntary reporting, which may introduce reporting bias and result in incomplete or inconsistent data. The reliance on spontaneous reporting means that adverse events, including postoperative dizziness, may be underreported or selectively reported, affecting the comprehensiveness of the findings. Second, due to the limitations of the FAERS database, we were unable to access detailed medical information, such as the patient’s surgical method, surgical site, duration of drug use, and other relevant health conditions that could influence the risk of postoperative dizziness. These factors, which are crucial in understanding the broader context of adverse drug reactions, were not available for analysis and may have contributed to unaccounted confounding in the study. Finally, while this study identified a significant association between certain drugs and postoperative dizziness, it is important to note that the findings are observational and do not establish causality. The study relies on existing reports, and the sample size may not be sufficient to draw definitive conclusions about the mechanisms behind the observed associations. Due to the limited sample size, we were unable to perform logistic regression analysis to adjust for confounding factors such as age, sex, comorbidities, and drug interactions. Therefore, further verification through prospective, controlled studies is needed to establish causal relationships and better understand the underlying factors that contribute to postoperative dizziness. Our findings have important implications for clinical practice, particularly in the context of postoperative analgesia and patient safety. The increased risk of dizziness in elderly patients, especially those receiving multiple medications, suggests a need for careful monitoring in this population. Clinicians should be particularly vigilant when prescribing drugs known to be associated with dizziness, especially in older patients who may already be at heightened risk due to age-related changes in physiology. Additionally, the potential for sex-based differences in dizziness risk calls for a more personalized approach to prescribing, where patient sex and age are considered as factors in drug selection. We recommend further studies that examine these demographic variables more closely, using larger and more diverse datasets, to establish whether gender-specific prescribing guidelines should be developed.

## Conclusion

In conclusion, our study provides valuable information on the trends and risk factors associated with dizziness in drug reports, offering a foundation for improving patient safety in clinical practice. However, further research is needed to explore these findings in greater depth, particularly with respect to age, sex, and medication interactions.

## Data Availability

The original contributions presented in the study are included in the article/supplementary material, further inquiries can be directed to the corresponding authors.
