# Corrigendum: Mining and analysis of dizziness adverse event signals in postoperative analgesia patients based on the FDA adverse event reporting system database

**DOI:** 10.3389/fphar.2025.1586073

**Published:** 2025-04-07

**Authors:** Fengqi Zhou, Haiou He, Jing Gao, Zhen Zhang

**Affiliations:** ^1^ Department of Anesthesiology, Xiangyang No. 1 People’s Hospital, Hubei University of Medicine, Xiangyang, Hubei, China; ^2^ Department of Anesthesiology, Suizhou Hospital, Hubei University of Medicine, Xiangyang, Hubei, China

**Keywords:** dizziness, postoperative analgesia, FAERS, drug safety, signal detection

In the published article, there was an error in the author order, and author Jing Gao was erroneously listed after author Zhen Zhang. The corrected author list appears below.

“Fengqi Zhou^1,†^, Haiou He^2,†^, Jing Gao^1,^*, Zhen Zhang^1,^*^”^.

The authors apologize for this error and state that this does not change the scientific conclusions of the article in any way. The original article has been updated.

